# BMI-Specific Nutritional Education Priorities for Weight Management in Osteoarthritis

**DOI:** 10.3390/nu17132056

**Published:** 2025-06-20

**Authors:** Ashley N. Buck, Danae C. Gross, Jieun (Jenna) Kim, Erica L. Rauff, Jennifer M. Dinallo, Lauren M. Abbate, Todd A. Schwartz, Nicholas J. Beresic, Connie B. Newman, Sarah P. Shultz

**Affiliations:** 1 Department of Exercise and Sport Science, University of North Carolina, Chapel Hill, NC 27599, USA; anbuck@unc.edu (A.N.B.); jenkim@unc.edu (J.K.); 2 Osteoarthritis Action Alliance, Chapel Hill, NC 27599, USA; dcgross@unc.edu (D.C.G.); lauren.abbate@va.gov (L.M.A.); cncbn@optonline.net (C.B.N.); 3 Thurston Arthritis Research Center, School of Medicine, University of North Carolina, Chapel Hill, NC 27599, USA; tschwart@email.unc.edu; 4 Human Movement Science Curriculum, School of Medicine, University of North Carolina, Chapel Hill, NC 27599, USA; 5 Department of Nutrition, University of North Carolina, Chapel Hill, NC 27599, USA; 6 Biobehavioral Health Department, Pennsylvania State University, University Park, PA 16802, USA; elr12@psu.edu (E.L.R.); jmd422@psu.edu (J.M.D.); 7 VA Eastern Colorado Geriatric Education and Clinical Center, Rocky Mountain Regional VA Medical Center, Aurora, CO 80045, USA; 8 Department of Biostatistics, University of North Carolina, Chapel Hill, NC 27599, USA; 9 Gillings School of Global Public Health, School of Medicine, University of North Carolina, Chapel Hill, NC 27599, USA; 10 Department of Medicine, Division of Endocrinology, Diabetes and Metabolism, New York University School of Medicine, New York, NY 10016, USA

**Keywords:** diet, obesity, body mass index

## Abstract

**Background/Objectives**: The educational needs of individuals with OA and obesity can drive personalized resources for effective dietary interventions that align patient interests with weight and disease management. Therefore, the purpose of the present study was to evaluate differences in nutritional education topics of interest between patients with OA who are characterized as having higher (≥30 kg/m^2^; HBMI) and lower BMI (<30 kg/m^2^; LBMI). **Methods**: Cross-sectional survey data (*n* = 296) were dichotomized into HBMI (*n* = 172; BMI: 38.67 ± 6.59 kg/m^2^) and LBMI (*n* = 124; BMI: 25.59 ± 3.00 kg/m^2^) groups. A mixed-method approach examined group differences across four primary domains: (i) strategies for weight management and healthy lifestyle, (ii) interest in vitamins and supplements, (iii) foods and nutrient with anti-inflammatory properties, and (iv) dietary patterns for weight loss. Logistic regression models compared topic interests between groups. Thematic analysis of open-ended responses captured additional insights. **Results**: Compared to LBMI participants, those in the HBMI group showed greater interest in weight loss strategies, emotional eating, and diets such as low-carbohydrate and ketogenic approaches, but less interest in general supplement information and plant-based diets. HBMI also had greater interest in practical strategies (e.g., feeling full, affordable foods) and reduced interest in certain anti-inflammatory foods. Both groups expressed a desire for evidence-based resources on foods that promote joint health. **Conclusions**: BMI-specific differences in nutritional education preferences highlight the need for tailored, patient-centered strategies in OA management. Addressing these differences may improve the effectiveness of education interventions and enhance patient–provider communication around diet and lifestyle in OA care.

## 1. Introduction

Osteoarthritis (OA) is a leading cause of global disability, affecting approximately 595 million individuals annually and imposing substantial personal and societal burdens [1]. Despite its prevalence and significant impact, no cure or effective disease-modifying treatments exist for OA, and thus symptom management is a primary treatment approach for OA management. As weight management is a key risk factor for OA onset and progression in both weight-bearing (e.g., knee, hip) and non-weight-bearing (e.g., hand) joints [2,3,4,5,6,7,8,9,10], weight loss and weight management strategies are critical for improving OA-related outcomes [5,6,7,8,10,11,12,13,14,15,16,17,18]. Additionally, recent meta-analyses demonstrate that individuals with OA have a 1.24-fold increased risk of cardiovascular disease and a 1.41-fold increased risk of diabetes mellitus compared to those without OA [19,20]. The high prevalence of these comorbid conditions among patients with OA and high BMI emphasizes the importance of lifestyle modifications such as healthy dietary patterns in addressing overlapping risk factors, particularly in this population [21,22,23]. Evidence highlights the benefits of regular physical activity and a healthy diet in weight loss and weight management plans, which often yield improved musculoskeletal pain, better physical function, decreased inflammation, and slower radiographic progression in OA [11,12,13,14,15,16,17,24]. Thus, developing patient-centered, evidence-based, and accessible interventions related to diet and exercise are critical in individuals with OA and obesity.

Individuals with OA express a strong interest in nutritional education to manage symptoms, reduce inflammation, and improve overall quality of life [25]. Patient education optimizes self-efficacy and facilitates adherence to interventions such as exercise and dietary changes, ultimately improving OA-related outcomes [26]. However, our previous work revealed that most healthcare professionals do not address nutrition education topics of interest with their patients [25]. Additionally, research has demonstrated significant disparities in access to OA management and education, with underserved populations often receiving limited guidance on lifestyle modifications [27,28]. Emphasizing whole foods and dietary patterns, rather than specific nutrients and supplements, may empower patients to adopt sustainable changes rather than face barriers related to cost, accessibility, and understanding of supplements for OA management.

Patient demographics, including age, sex, race, and particularly body mass index (BMI), significantly influence OA-related outcomes and adherence to lifestyle modifications. Psychosocial barriers, such as emotional eating and low self-efficacy, disproportionately affect patients with high BMI [11] and highlight the importance of targeted education to support diet and exercise modification. Specifically, emotional eating has been linked to depressive symptoms and greater consumption of energy-dense foods, while low self-efficacy is associated with reduced physical function and poorer engagement in self-management behaviors [29,30,31]. These barriers exacerbate disease progression and require tailored approaches that consider demographic factors, such as BMI, to ensure equitable care. Access to OA-related healthcare, type of OA management, and cost of OA-related healthcare is also found to differ between those with overweight and obesity class [32]. Despite the influence of BMI on critical factors linked to OA-related outcomes and management, limited research has directly compared the educational needs of patients across various BMI categories. This gap hinders the development of effective, patient-centered interventions that align healthcare management with patient-specific interests and needs.

Examining the educational needs of individuals with OA and obesity can aid in developing personalized resources for effective dietary interventions that align with patient interests. Subsequently, effectiveness of patient–provider interactions in OA management could be improved by considering BMI-specific nutritional education priorities in those with OA. Thus, the primary purpose of the present study was to evaluate differences in nutritional education topics of interest between patients with OA who are characterized within our study sample as having a higher BMI (≥30 kg/m^2^; HBMI) and those within our cohort with lower BMI (<30 kg/m^2^; LBMI). Through a combination of (i) quantitative and (ii) qualitative analyses, we hypothesized that significant differences in patients’ interests in nutrition and lifestyle education topics/themes will emerge between dichotomous BMI sub-groups of our study sample, thereby serving as an initial step for providing a foundation to develop and optimize effective personalized patient education strategies for OA management.

## 2. Materials and Methods

### 2.1. Study Design

We conducted a secondary data analysis of survey data originally collected between July 2020 and February 2021, the development of which has been previously described [25]. The survey was a cross-sectional, anonymous, electronic questionnaire focused on nutrition education topics for OA management. The survey was distributed to both individuals with OA and healthcare professionals who treat patients with OA. The initial study [25] examined (1) key nutrition education topics of interest for patients with OA, (2) patients’ preferred modalities for delivering nutrition education information, and (3) nutrition education topics that healthcare professionals report discussing with their patients. The present analyses sought to expand upon our original findings by evaluating whether nutritional education topics of interest differed between individuals in our cohort with a higher BMI (≥30 kg/m^2^; HBMI) and those in our study with a lower BMI (<30 kg/m^2^; LBMI). To examine differences in nutritional topics of interest between patients with lower and higher BMI, only responses from the patient survey were included; responses from the healthcare professionals survey were not linked to specific patients and therefore were not relevant to the present analyses.

This study was approved by the University of North Carolina at Chapel Hill Institutional Review Board (IRB #20-199) and was deemed exempt from federal human subject research regulations. All participants provided written, informed consent prior to study participation. No personal identifying information was collected, and all survey data were securely stored on a UNC-approved secure server. Data access was restricted to authorized study personnel using institutional credentials and two-factor authentication.

### 2.2. Participants and Recruitment

Patients with OA were recruited using a combination of digital and organizational channels, including social media platforms (e.g., Facebook and X, formerly Twitter), the Osteoarthritis Action Alliance (OAAA) website, and ResearchMatch (a national health volunteer registry). Recruitment materials and study flyers were distributed widely through OAAA’s communication networks and partnerships.

Eligibility criteria required participants to be at least 18 years of age, reside in the United States, and self-report a diagnosis of hip or knee OA. Survey responses were excluded if: (i) the participant resided outside the United States, (ii) participants did not respond to at least one survey question, or (iii) they did not have a self-reported diagnosis of hip or knee OA [25]. Additionally, responses with incomplete self-reported height and weight data were excluded from the present analysis, as BMI could not be calculated. Previous research has found that using self-reported height and weight to calculate BMI is a valid approach [33]. Participation in the survey was voluntary, and no monetary or other incentives were provided.

The original study [25] included data from 338 individuals with OA, while the present study included data from 296 patient responses. Forty-two survey responses were excluded in these analyses due to incomplete height or weight data. To assess whether missing or incomplete data were missing at random, we compared the distribution of descriptive characteristics (e.g., age, sex, race, education) between surveys with missing data and the entire cohort. No significant differences were observed within the Interquartile Range distribution between survey demographics with missing data and the entire cohort, thereby indicating that data were missing at random.

### 2.3. Patient Survey Content

The survey for individuals with OA was designed to evaluate participants’ nutritional education priorities and their preferred delivery methods to receive nutrition education [25]. The survey consisted of multiple response and open-ended questions across four primary domains: (i) strategies for weight management and healthy lifestyle, (ii) interest in vitamins and supplements, (iii) foods and nutrient with anti-inflammatory properties, and (iv) dietary patterns for weight loss. For each multiple response question, participants selected at least 1 topic of interest within each domain and had the option to select their preferred 3–5 nutritional topics of interest within each domain. Each section also included an open-ended question to capture additional topics of interest that were not explicitly identified as survey responses. Demographic data including age, sex, height, weight, race, and education status were collected to contextualize the cohort and assess variations in nutritional topics of interest across subgroups. This structured approach ensured that the survey captured actionable insights into patient preferences while maintaining clarity, accessibility, and translatability.

### 2.4. Data Handling

Survey data were collected using Qualtrics (Qualtrics, LLC; Provo, UT, USA). Data access was restricted to authorized study personnel using institutional credentials and two-factor authentication. Survey responses were anonymized, and no personal identifiers were collected. Participants had the option to provide their email addresses for future communication about OA-related resources; these emails were collected via a separate form and stored independently from survey responses in a password-protected file. All data were stored on the source OAAA drive which is maintained on a UNC-approved secure server. Data analysis was conducted using the built-in reporting features on Qualtrics, and datasets were exported as CSV files for further statistical analyses.

### 2.5. Quantitative Statistical Analyses

All quantitative statistical analyses were conducted in Python (v.3.12; Python Software Foundation, Wilmington, DE, USA). BMI was calculated from self-reported height and weight; the sample size restricted equitable distribution of BMI across standard classifications. Therefore, participants were allocated to one of two groups, based on the BMI distribution of our study sample: (i) HBMI (≥30 kg/m^2^) or (ii) LBMI (<30 kg/m^2^). BMI was evaluated both categorically using the dichotomous cut-off point of 30 kg/m^2^ as well as continuously to account for variation within groups. Data from multiple response questions were dichotomized by recoding each possible response as binary yes (1) or no (0). A response coded as “yes” or “1” indicated that the participant selected that option within the multiple response question as a topic of interest for the corresponding question, while “no” or “0” indicated that the patient did not select that topic of interest for the question. Sex and race were also dichotomized such that “0” indicated male or white, and “1” indicated female or other races, respectively. Normality of the data was evaluated using Shapiro–Wilk tests and visually inspecting Q-Q plots. Between group comparisons were conducted to determine whether descriptive outcomes (e.g., age, sex, race, education status) differed between the BMI groups.

Separate logistic regression models evaluated the associations between dichotomous BMI and patient-reported interest for each nutrition education topic. Corresponding odds ratios (OR) were calculated from these models to determine increased or decreased interest in a nutrition education topic of interest based on BMI. Secondary analysis included logistic regression models that used continuous BMI values as the predictor variable to further contextualize the relationship between a 1 kg/m^2^ unit increase in BMI beyond 30 kg/m^2^ and the odds of reporting interest in a nutrition education topic. As demographic variables such as age, sex, and race did not differ between BMI groups, the primary logistic regression models were unadjusted. However, to further contextualize and understand the influence of BMI alone on nutrition education topics of interest, additional adjusted logistic regression models controlled for age (continuous variable), sex (male as the referent), and race (white as the referent).

### 2.6. Qualitative Statistical Analyses

Two members of the research team reviewed and coded the qualitative responses to six open-ended questions from the survey. The first four questions provided optional open-ended responses when participants were asked to choose which topics they prioritized around dietary themes related to joint health: (1) strategies for weight management and healthy lifestyle; (2) vitamins, minerals, and other supplements; (3) foods and nutrients that may reduce inflammation; and (4) diets for weight loss. The final two questions asked participants to: (5) list other nutrition topics related to joint health they would like to learn about; and (6) specify other resources about joint health and nutrition they would like to learn about. Principles of thematic analysis were used to analyze the qualitative responses [34]. Themes that emerge from qualitative analysis reflect a range of individual attitudes, opinions, and beliefs that are grouped together based on similarity into a coding scheme developed by the research team [35]. To achieve this goal, responses were coded individually by each researcher using a thematic framework that was developed inductively through which each individual code was categorized into a higher order theme. For instance, the first question asked participants what information they would like regarding weight management and healthy lifestyles and three participants responded with the following comments “binge eating disorder,” “treatment for eating disorders,” and “treatment for eating disorders.” Each of these individual responses were coded under the higher order theme of treatment for eating disorders. Each researcher coded responses into themes individually and maintained their own codebooks. Both researchers met three times to review code books and ensure 100% agreement on the coding structure. When disagreement in coding was found, the researchers discussed the rationale for their coding, reviewed participants’ responses again for further clarity and discussed the appropriate code that should be given to each response. A log was kept of any code in which there was disagreement in which the researchers logged discussions around the coding of the response, changes that would be made to the code, and the final decision that was made for the coding of that response. Each question resulted in a varying number of themes, which was driven by the number of responses participants provided for each question. In some instances, participant responses for a specific question were irrelevant (i.e., did not qualify as an answer to the question asked) and excluded (*n* = 15 individual responses across the 6 questions for both BMI groups). Some responses were not relevant to the question being asked but answered a different question and were coded with the relevant question instead (*n* = 6 individual responses across the 6 questions for both BMI groups). Some responses were not relevant to the topic of nutrition but did provide context regarding information participants wanted on other topics related to joint health (*n* = 25 individual responses across the 6 questions for both BMI groups). In the cases in which codes were moved to other questions or categorized as irrelevant, the two researchers completed the same process as described above, which included individual coding and consensus meetings to determine whether the responses given by participants were relevant to the question being asked. If the response was deemed as irrelevant to the question prompt, it was either categorized as irrelevant or moved to a different question prompt that was determined by both researchers to more accurately respond to the question being asked.

## 3. Results

Demographic data for the entire group as well as each BMI subgroup are presented in Table 1. Data from 296 individuals were included in the present analyses. A total of 172 individuals (58.10%) were dichotomized to the HBMI group and the remaining 124 individuals were classified as having LBMI. No between-group differences were found between the HBMI and LBMI groups for age, sex, and race (Table 1). Unadjusted and adjusted (age, sex, and race) ORs and their corresponding 95% CIs for each model (using dichotomous BMI and continuous BMI as explanatory variables) are presented in Table 2.

### 3.1. Strategies for Weight Management and a Healthy Lifestyle

**Figure 1:**
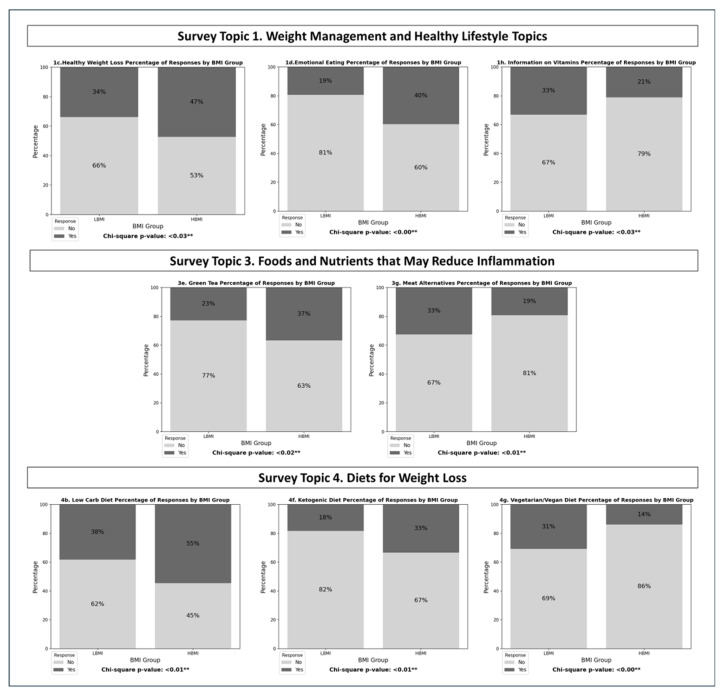
Bar graphs and unadjusted logistic regression *p*-value results for survey questions that represent a statistically significant result from the unadjusted logistic regression models with dichotomous BMI as the explanatory variable. Statistically significant results are based on dichotomous BMI subgroup (lower BMI: <30 kg/m^2^; higher BMI: ≥30 kg/m^2^). All data (regardless of statistical significance) are visually represented in Supplementary Materials. ** denotes statistical significance.

Compared to the LBMI group, individuals within HBMI group demonstrate significantly (*p* < 0.05) greater odds of interest in (a) healthy weight loss and (b) ways to help control emotional eating. Further, compared with LBMI, HBMI demonstrated less interest in learning about general information on vitamins, minerals, supplements (Figure 1; Table 3).

**Figure 2:**
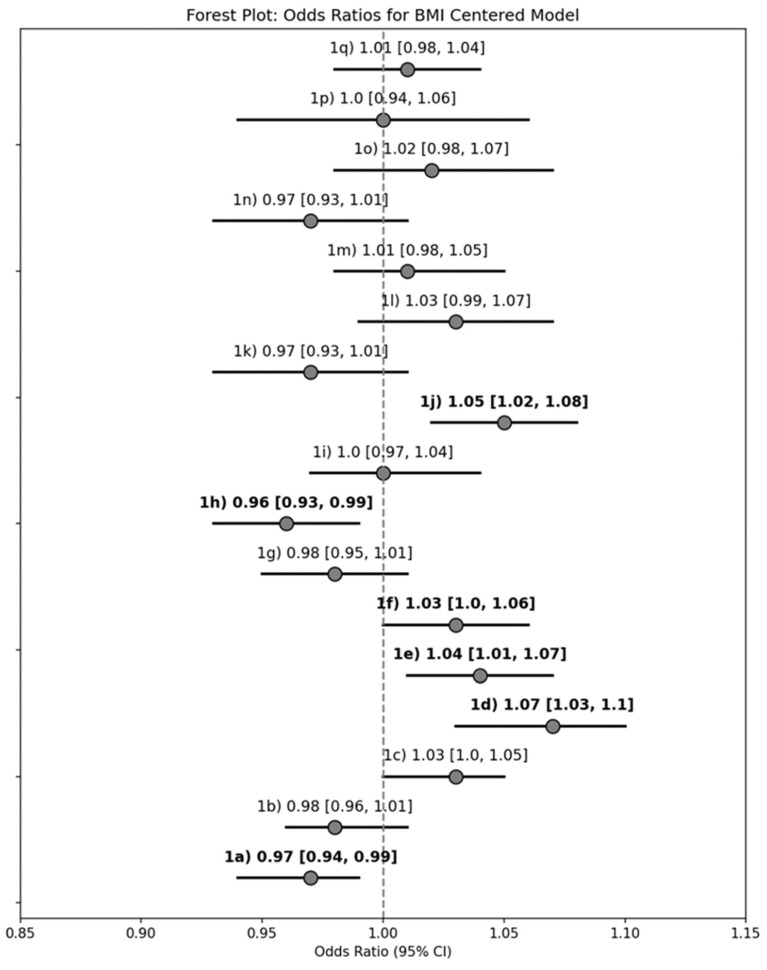
Forest plot summary of the unadjusted logistic regression models with continuous BMI as the explanatory variable for determining the odds of reporting interest in learning about each Strategy for Weight Management and a Healthy Lifestyle topic of interest. Results reported as odds ratio [95% confidence interval], and bold text indicates a statistically significant model (95% CI does not span 0).

For every 1 kg/m^2^ increase in BMI, there were significantly greater odds of reporting interest in (a) ways to control emotional eating (unadjusted OR: 1.07 [95%CI: 1.03–1.10]), (b) strategies to feel full (1.04 [1.01–1.07]), (c) affordable food choices (1.03 [1.00–1.06]), and (d) diet considerations for other health conditions (1.05 [1.02–1.08]). Additionally, there were decreased odds of reporting interest in (e) foods that make OA symptoms worse (0.97 [0.94–0.99]) and (f) general information on vitamins, minerals, and supplements (0.95 [0.93–0.99]; Figure 2).

Qualitatively, participants in LBMI reported wanting information about how to reduce sugar cravings, foods with anti-inflammatory properties, alcohol and osteoarthritis, and easy meals for increasing energy (Table 4). Participants in HBMI reported wanting easy to prepare meals, how to lose weight with multiple health issues, and medication that causes weight gain. Three participants within HBMI indicated wanting information about treatment for eating disorders, which was a response not identified in LBMI group.

### 3.2. Vitamins, Minerals and Other Supplements

There were no significant differences (*p* > 0.05) between LBMI and HBMI when reporting interest in learning about vitamins, minerals, and other supplements (Figure S2). Similarly, there were no significant changes (*p* > 0.05) in the odds of reporting interest in learning about topics of vitamins, minerals, and other supplements for each 1 kg/m^2^ increase in BMI beyond 30 kg/m^2^ (Figure S3).

Participants in LBMI most commonly reported wanting information about cannabis-derived compounds (i.e., CBD, THC), which was not reported by any participants within HBMI (Table 4). HBMI participants most commonly reported wanting information about (a) B vitamins and (b) over the counter pain relief. They also reported wanting additional information regarding supplements such as turmeric and minerals (i.e., zinc and calcium). Both groups of participants wanted information about collagen and hyaluronic acid.

### 3.3. Foods and Nutrients That May Reduce Inflammation

Compared with LBMI, a significantly (*p* < 0.05) higher proportion of HBMI participants report interest in learning about green tea, and a significantly lower proportion report interest in learning about plant-based alternatives for meat (Figure 1; Table 3).

**Figure 3:**
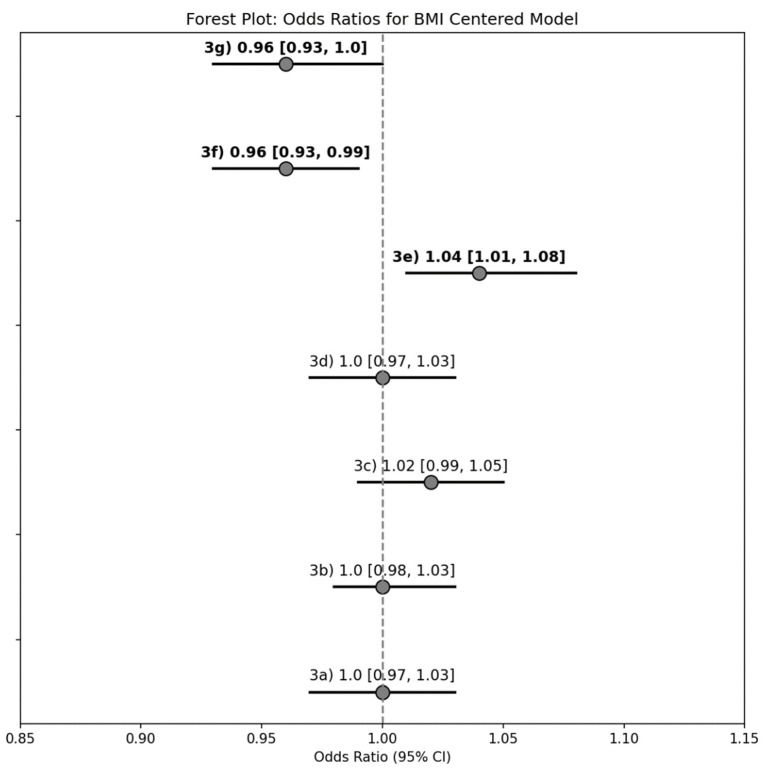
Forest plot summary of the unadjusted logistic regression models with continuous BMI as the explanatory variable for determining the odds of reporting interest in learning about Foods and Nutrients that may Reduce Inflammation topics of interest. Results reported as odds ratio [95% confidence interval], and bold text indicates a statistically significant model (95% CI does not span 0).

For every 1 kg/m^2^ increase in BMI, there were increased odds of reporting interest in learning about (a) green tea (1.04 [1.01–1.08]), and decreased odds of reporting interest in learning about (b) fish oil (0.96 [0.93–0.99]) and (c) plant-based meat alternatives (0.96 [0.93, 1.00]; Figure 3).

When asked about information they would like regarding food and nutrients that may reduce inflammation, participants within LBMI only provided responses about chocolate (*n* = 1) and alternative foods for those with intestinal issues (*n* = 1). HBMI participants also provided limited responses for this question but reported that they would like information about butter substitutes, shellfish, and tart cherries (Table 4).

### 3.4. Diets for Weight Loss

Compared with LBMI, a significantly (*p* < 0.05) higher proportion of individuals within HBMI report interest in learning about (a) low carbohydrate diet and (b) ketogenic diet, along with a significantly lower proportion reporting interest in the (c) vegetarian and vegan diets (Figure 1; Table 3).

**Figure 4:**
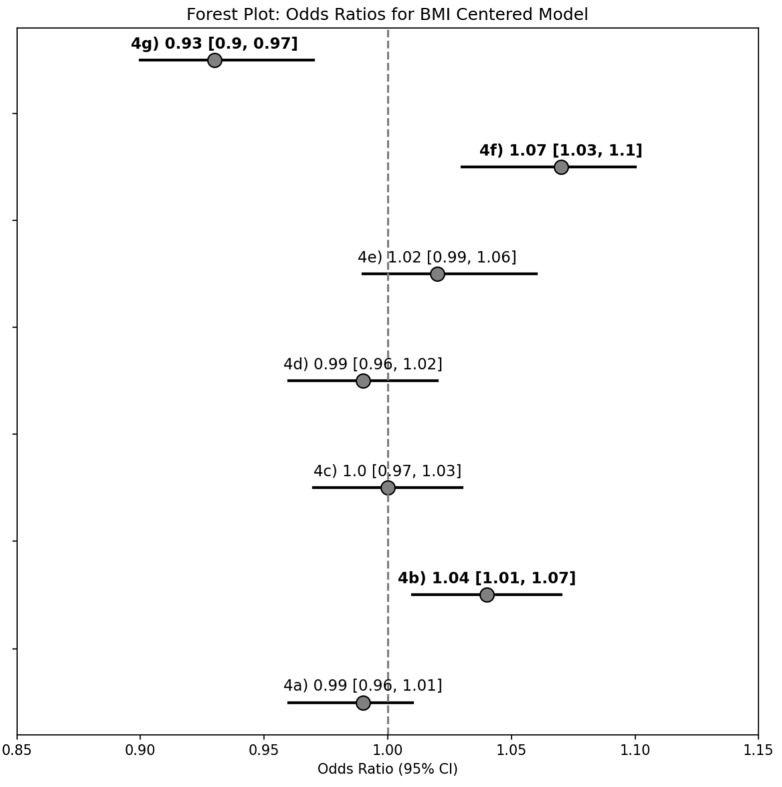
Forest plot summary of the unadjusted logistic regression models with continuous BMI as the explanatory variable for determining the odds of reporting interest in learning about Diets for Weight Loss topics of interest. Results reported as odds ratio [95% confidence interval], and bold text indicates a statistically significant model (95% CI does not span 0).

For every 1 kg/m^2^ unit increase in BMI beyond 30 kg/m^2^, there were increased odds of reporting interest in (a) low carbohydrate diet (1.04 [1.01–1.07]) and (b) ketogenic diet (1.07 [1.03–1.10]), along with decreased odds of reporting interest in vegetarian/vegan diets (0.93 [0.90–0.97]; Figure 4).

Participants in LBMI provided limited responses regarding diets for weight loss, suggesting they would like information about MIND (Mediterranean-DASH Intervention for Neurodegenerative Delay) diet (*n* = 1), Weight Watchers (*n* = 1), and diets to reduce sugar (*n* = 1). However, HBMI participants provided more responses to this question with three participants indicating they’d like information about diets to improve health conditions, three participants wanting information about balanced eating plans, and one participant requesting Southern recipes (Table 4).

### 3.5. Resources and Additional Nutrition Topics

Participants from both BMI groups commonly reported wanting to know more about foods to manage inflammation and pain (Table 4). The other most salient response for LBMI regarding other nutrition topics was information for diets for joint health for those who also have other chronic health conditions such as irritable bowel syndrome (IBS) or gastroesophageal reflux disease (GERD). HBMI participants commonly reported wanting other nutrition information on (a) specific foods for joint health (i.e., Asian diet, bone broth, white flour, Coke, and diet colas), (b) foods that contribute to unhealthy weight and increased pain, (c) foods to prevent joint deterioration or improve joint health, and (d) nutritional choices to improve bone density and prevent fractures. Finally, both groups commonly reported wanting resources that were evidence-based. LBMI participants reported desiring resources more broadly such as podcasts or audio books. HBMI participants wanted more hands-on resources such as appointments with specialists (i.e., rheumatologists, gastric-bypass specialists, eating disorder therapists, nutritionists, and support groups) and information on how to participate in research studies.

## 4. Discussion

Patient preferences for osteoarthritis treatment methods and educational materials is consistent with patient-centered medicine and may improve adherence to therapies [36]. This study aimed to provide greater insight on the educational and nutrition topics of interest for patients with OA by dichotomous BMI subgroups (<30 kg/m^2^ vs. ≥30 kg/m^2^). Generally, individuals within HBMI group expressed a greater interest in strategies for healthy weight loss and controlling emotional eating, while indicating less interest in general information about vitamins, minerals, and supplements. In contrast, individuals within LBMI were more interested in topics such as reducing sugar cravings, anti-inflammatory foods, and interrelationships between OA and recreational substance (i.e., alcohol, THC, CBD). There were some similarities between groups, primarily regarding their desire for evidence-based resources on foods that promote joint health and help manage their OA.

Individuals within HBMI showed significant interest in the psychosocial component of healthy eating, with a stronger desire to understand how to control emotional eating and feeling full. Emotional eating includes behaviors where individuals eat in response to negative emotions; this behavior has a positive relationship with weight gain and difficulty losing weight [37,38]. Conversely, intuitive and mindful eating focuses on an individual listening to their own hunger, fullness, and taste satiety cues [38,39]. In one systematic review, mindful and intuitive eating approaches have demonstrated modest but statistically significant weight loss when compared to no intervention at all [40]; their effectiveness appears comparable to that of conventional diet programs. While not considered an eating disorder, emotional eating is correlated to depression, anxiety, and stress [29,36]. However, our qualitative findings support these interrelationships, where individuals with a higher BMI also prioritize information regarding treatment for eating disorders.

Individuals within HBMI showed greater interest in weight loss rather than weight maintenance, with interests in consuming fewer carbohydrates and creating balanced eating plans. Higher BMI is oftentimes attributed to the cumulative effects of excessive intake and sedentary lifestyle. While excessive caloric intake and decreased nutritional value of foods consumed are attributed to higher BMI and subsequent obesity-related outcomes, there are also underlying physiological mechanisms which contribute to caloric intake and types of food consumed [41]. It is well known within the literature that large, processed meals with servings high in refined grains, red meat, saturated fats, and added sugars contribute to increased adiposity and weight gain [42]. However, improving nutritional patterns and intake is considered an effective strategy to mitigate inflammatory burden and diet-related diseases. Foods with anti-inflammatory properties can promote healthy weight, such as whole grains, vegetables, fruits, and nuts [43]. The Mediterranean Diet emphasizes a larger intake of fruits and vegetables and has been linked to increased bone health and physical function, while protecting against muscle wasting [44]. The HBMI group had little interest in vegetarian/vegan diets or learning about plant-based alternatives for meat. Some data suggest that plant-based diets may be more beneficial for weight loss and metabolic outcomes [45]; however, there is limited data in this field and future research should determine the effects of plant-based diets on OA outcomes.

All participants showed significant interest in diets for joint health, reducing inflammation, and decreasing joint pain. There is strong evidence in the literature suggesting that a dietary and exercise intervention approach for weight loss and OA management optimizes OA outcomes compared with diet or exercise alone [5,6,7,8,9]. Further, most evidence in previous literature supports the benefits of physical activity and exercise for OA management [5,6,7,8,9,10,18], with less evidence and research in understanding dietary patterns for optimizing biological and patient-reported OA outcomes [46]. Thus, most evidence-based guidelines for OA treatment largely focus on exercise and place less emphasis on dietary modifications [11,47,48]. Our recent umbrella review highlighted the need for diet research that identifies consumer-accessible foods for OA management [46]. Thus, future work should seek to understand the interrelationships between dietary patterns and OA outcomes to inform evidence-based resources for patients which they are interested in learning about.

There is a strong desire for all participants to have evidence-based resources so that they can make informed decisions regarding their diets and eating behaviors. Individuals in LBMI preferred self-directed resources while the HBMI group showed more interest in hands-on approaches to weight loss with interest in meetings with other specialists (e.g., gastric bypass) and participation in research studies. It is important to note that health care professionals have reported discussing weight loss strategies with patients but report some reluctance to provide recommendations on dietary patterns that can manage weight and OA symptoms without conclusive data to support the guidance [25]. Thus, regardless of BMI, there is tension between the information that patients with OA request and the ability to provide evidence-based recommendations.

This study is not without limitations. As mentioned in our original study [25], there might be self-selection bias in individuals with OA who responded to the survey. These individuals are clear in their symptoms and pro-active in their management, which can impact their approach to manage and learn about OA symptoms. Along with self-reporting their OA diagnosis, individuals were also asked to self-report height and weight to calculate BMI. While this approach has been validated in previous research [33] to determine obesity in population-based studies, there have been some conflicting results concerning underestimation of BMI [49,50]. Particularly, older adults can inaccurately report height because of unnoticed shifts in stature as they aged. However, the potential inaccuracy should have minor impact to our findings as the difference between groups in the current study is well beyond the previously reported difference of approximately 2 kg/m^2^. Additionally, this study had a higher proportion of female participants compared to male participants. While sex distribution was even across groups and should not impact group comparisons, the oversampling may impact the generalizability of our findings. Finally, research has indicated that comorbid conditions are of a greater prevalence in patients with OA and obesity, yet we did not account for the impact these comorbidities might have on an individual’s nutritional preferences. This complex interaction between conditions and nutrition preference warrants further investigation.

## 5. Conclusions

This study identified critical differences in OA patients’ preferences for nutrition education and healthy lifestyle topics between those characterized within our cohort as having a lower BMI (<30 kg/m^2^) vs. a higher BMI (≥30 mg/kg^2^). Our results suggest that more tailored and patient-specific approaches may be necessary to optimize OA and weight management and better support individuals living with OA. While weight management has frequently incorporated lifestyle and behavior modifications, there is a gap in research evaluating OA self-management topics of interest from a similar perspective. Hence, our data are among the first to assess holistic patient interests in OA and weight management and to identify critical differences in patient preferences between BMI groups. The findings of this study, along with the scarcity of similar research, underscore the need for further exploration of patient preferences at the intersection of OA and weight management to inform precision medicine interventions. Understanding these preferences is essential for developing and implementing effective, patient-centered strategies that optimize OA and weight management and reduce their burden for individuals living with osteoarthritis and obesity.

## Figures and Tables

**Table 1:** Participant characteristics and demographics for the entire cohort (*n* = 296) and each body mass index (BMI) subgroup (Higher BMI, ≥30 kg/m^2^; Lower BMI, <30 kg/m^2^). Data are presented as mean ± standard deviation or *n* (%), as appropriate.

	Entire Study Sample	BMI: ≥30 kg/m^2^	BMI: <30 kg/m^2^	*p*-Value
***n* (%)**	296 (100%)	172 (58.10%)	124 (41.89%)	
**Age**	60.30 ± 10.80	58.93 ± 10.55	62.24 ± 10.91	0.06
**BMI**	33.19 ± 8.41	38.67 ± 6.59	25.59 ± 3.00	<0.001
**Sex**				0.35
Male, *n* (%)	47 (15.88%)	25 (14.53%)	22 (17.74%)	
Female, *n* (%)	249 (84.12%)	147 (85.47%)	102 (82.26%)	
**Race**				0.07
Caucasian, *n* (%)	253 (85.47%)	139 (80.81%)	114 (91.94%)	
Other Races, *n* (%)	43 (14.53%)	33 (19.19%)	10 (8.06%)	
**US Region**				
Atlantic	112 (37.84%)	61 (35.47%)	51 (41.13%)	
Southern	63 (21.28%	37 (21.51%)	26 (20.97%)	
Central	66 (22.30%)	44 (25.58%)	22 (17.74%)	
Pacific	49 (16.55%)	28 (16.28%)	21 (16.94%)	

Notes: US regions were divided into patients residing in Atlantic (Maine, Massachusetts, New Hampshire, Connecticut, New York, New Jersey, Pennsylvania, Delaware, Maryland, Virginia, North Carolina), Southern (South Carolina, Georgia, Florida, Tennessee, Alabama, Mississippi, Arkansas, Oklahoma, Texas), Central (West Virginia, Ohio, Kentucky, Indiana, Illinois, Michigan, Wisconsin, Minnesota, Iowa, Missouri, Kansas, Nebraska, South Dakota), and Pacific (Montana, Colorado, Arizona, Nevada, California, Oregon, Washington, Hawaii) states.

**Table 2:** Summary of unadjusted and adjusted logistic regression odds for dichotomous and continuous BMI predictors for each survey question response.

Survey Topic and Question	Logistic Models with Dichotomous BMI Predictor ^a^		Logistic Models Continuous BMI Predictor ^b^	
**1. Weight Management and Healthy Lifestyle Topics**	Unadjusted	Adjusted ^c^	Unadjusted	Adjusted ^c^
	OR (95% CI)	OR (95% CI)	OR (95% CI)	OR (95% CI)
1a. Foods that make OA symptoms worse	0.69 (0.42, 1.13)	0.66 (0.39, 1.13)	**0.97 (0.94, 0.99)**	**0.96 (0.92, 0.99)**
1b. Foods to help reduce inflammation	0.66 (0.41, 1.05)	0.73 (0.44, 1.22)	0.98 (0.96, 1.01)	0.98 (0.96, 1.01)
1c. Healthy weight loss for older adults	**1.74 (1.08, 2.80)**	**2.64 (1.52, 4.58)**	1.03 (1.00, 1.05)	**1.05 (1.02, 1.08)**
1d. Ways to control emotional eating	**2.72 (1.59, 4.68)**	**2.39 (1.35, 4.23)**	**1.07 (1.03, 1.10)**	**1.06 (1.03, 1.10)**
1e. Strategies to feel full	1.47 (0.89, 2.42)	1.62 (0.94, 2.78)	**1.04 (1.01, 1.07)**	**1.04 (1.01, 1.08)**
1f. Information on affordable food options	1.47 (0.89, 2.42)	1.42 (0.84, 2.42)	1.03 (1.00, 1.06)	1.03 (1.00, 1.06)
1g. Tips for healthy snacking	0.78 (0.47, 1.28)	0.83 (0.49, 1.42)	0.98 (0.95, 1.01)	0.98 (0.95, 1.01)
1h. General info on vitamins and minerals	**0.54 (0.32, 0.91)**	**0.54 (0.30, 0.94)**	**0.96 (0.93, 0.99)**	**0.96 (0.92, 0.99)**
1i. Awareness of food triggers	1.06 (0.63, 1.81)	0.99 (0.55, 1.76)	1.00 (0.97, 1.04)	1.00 (0.96, 1.03)
1j. Special diet considerations	1.70 (0.97, 2.98)	**1.95 (1.05, 3.62)**	**1.05 (1.02, 1.08)**	**1.06 (1.02, 1.10)**
1k. Mindful eating techniques	0.64 (0.35, 1.16)	0.52 (0.27, 1.02)	0.97 (0.93, 1.01)	**0.96 (0.92, 1.00)**
1l. Ways to monitor/control caloric intake	1.60 (0.82, 3.11)	1.66 (0.80, 3.44)	1.03 (0.99, 1.07)	1.03 (0.99, 1.07)
1m. Benefits to fasting/intermittent fasting	1.67 (0.89, 3.13)	1.40 (0.71, 2.76)	1.01 (0.98, 1.05)	1.00 (0.96, 1.04)
1n. Impacts of organic vs. regular food	0.54 (0.28, 1.06)	0.60 (0.29, 1.21)	0.97 (0.93, 1.01)	0.97 (0.93, 1.02)
1o. Ways to control portion size	1.25 (0.60, 2.59)	1.33 (0.58, 3.02)	1.02 (0.98, 1.07)	1.05 (1.00, 1.10)
1p. Food labels to guide dietary decisions	1.47 (0.49, 4.41)	1.34 (0.43, 4.18)	1.00 (0.94, 1.06)	0.99 (0.93, 1.05)
**2. Vitamins, Minerals, and Supplements**	Unadjusted	Adjusted ^c^	Unadjusted	Adjusted ^c^
	OR (95% CI)	OR (95% CI)	OR (95% CI)	OR (95% CI)
2a. Glucosamine	1.53 (0.94, 2.49)	1.43 (0.86, 2.36)	1.02 (0.99, 1.05)	1.01 (0.98, 1.04)
2b. Vitamin D	0.84 (0.52, 1.36)	0.83 (0.50, 1.36)	0.98 (0.96, 1.01)	0.98 (0.95, 1.01)
2c. Omega 3 Fatty Acids	0.88 (0.55, 1.44)	0.92 (0.56, 1.52)	0.98 (0.96, 1.01)	0.99 (0.96, 1.02)
2d. Calcium	0.87 (0.52, 1.42)	0.87 (0.52, 1.45)	0.99 (0.96, 1.02)	0.99 (0.96, 1.02)
2e. Chondroitin	1.22 (0.74, 2.02)	1.29 (0.77, 2.18)	1.03 (1.00, 1.06)	**1.03 (1.00, 1.06)**
2f. Amino Acids	0.71 (0.41, 1.21)	0.70 (0.40, 1.22)	1.00 (0.97, 1.03)	1.00 (0.97, 1.03)
2g. Dietary Fiber	0.94 (0.53, 1.65)	0.98 (0.54, 1.75)	1.01 (0.98, 1.04)	1.01 (0.98, 1.05)
**3. Foods and Nutrients that may Reduce Inflammation**	Unadjusted	Adjusted ^c^	Unadjusted	Adjusted ^c^
	OR (95% CI)	OR (95% CI)	OR (95% CI)	OR (95% CI)
3a. Spices and Herbs	0.77 (0.45, 1.29)	0.90 (0.52, 1.54)	1.00 (0.97, 1.03)	1.01 (0.98, 1.04)
3b. Fruits and Vegetables	1.27 (0.78, 2.08)	1.28 (0.77, 2.12)	1.00 (0.98, 1.03)	1.01 (0.98, 1.04)
3c. Nuts	1.38 (0.84, 2.26)	1.39 (0.84, 2.32)	1.02 (0.99, 1.05)	1.03 (1.00, 1.06)
3d. Olive Oil	1.08 (0.64, 1.80)	1.15 (0.68, 1.94)	1.00 (0.97, 1.03)	1.00 (0.97, 1.03)
3e. Green Tea	**2.01 (1.17, 3.47)**	**1.71 (1.00, 3.00)**	**1.04 (1.01, 1.08)**	1.03 (1.00, 1.06)
3f. Fish Oil	0.62 (0.35, 1.08)	0.65 (0.37, 1.16)	**0.96 (0.93, 0.99)**	**0.96 (0.92, 1.00)**
3g. Plant-Based Alternatives for Meat	**0.41 (0.24, 0.72)**	**0.38 (0.21, 0.68)**	**0.96 (0.93, 1.00)**	**0.95 (0.91, 0.98)**
**4. Diets for Weight Loss**	Unadjusted	Adjusted ^c^	Unadjusted	Adjusted ^c^
	OR (95% CI)	OR (95% CI)	OR (95% CI)	OR (95% CI)
4a. Mediterranean Diet	0.76 (0.47, 1.24)	0.82 (0.49, 1.36)	0.99 (0.96, 1.01)	0.99 (0.96, 1.02)
4b. Low Carbohydrate Diet	**1.92 (1.17, 3.15)**	**1.92 (1.15, 3.19)**	**1.04 (1.01, 1.07)**	**1.04 (1.01, 1.08)**
4c. Fasting and Intermittent Fasting	1.03 (0.63, 1.68)	0.90 (0.54, 1.51)	1.00 (0.97, 1.03)	0.99 (0.97, 1.03)
4d. High Protein Diet	1.03 (0.63, 1.70)	1.03 (0.61, 1.73)	0.99 (0.97, 1.02)	0.99 (0.96, 1.02)
4e. DASH Diet	1.42 (0.82, 2.44)	1.61 (0.91, 2.87)	1.02 (0.99, 1.06)	1.03 (1.00, 1.07)
4f. Ketogenic Diet	**2.30 (1.29, 4.09)**	**2.05 (1.13, 3.70)**	**1.07 (1.03, 1.10)**	**1.06 (1.02, 1.10)**
4g. Vegetarian or Vegan Diets	**0.38 (0.21, 0.69)**	**0.40 (0.22, 0.74)**	**0.94 (0.90, 0.98)**	**0.94 (0.90, 0.98)**

Notes: ^a^ dichotomous BMI cutoff = 30 kg/m^2^; ^b^ continuous BMI prediction models centered at 30 kg/m^2^; ^c^ adjusted models controlling for age, male sex, and white race; bold values represent statistically significant model (*p* < 0.05); OR, odds ratio; CI, confidence interval.

**Table 3:** Distribution of patient responses by group for each survey question.

Survey Topic and Question	LBMI		HBMI		*p*-Value
1. Weight Management and Healthy Lifestyle Topics	*n* = “No”	*n* = “Yes”	*n* = “No”	*n* = “Yes”	
1a. Foods that make OA symptoms worse	38	86	67	105	0.177
1b. Foods to help reduce inflammation	45	79	80	92	0.102
1c. Healthy weight loss for older adults	82	42	91	81	**0.031**
1d. Ways to control emotional eating	100	24	104	68	**0.000**
1e. Strategies to feel full	89	35	109	63	0.164
1f. Information on affordable food options	89	35	109	63	0.164
1g. Tips for healthy snacking	82	42	123	49	0.388
1h. General info on vitamins and minerals	83	41	136	36	**0.027**
1i. Awareness of food triggers	93	31	127	45	0.927
1j. Special diet considerations	101	23	124	48	0.085
1k. Mindful eating techniques	97	27	146	26	0.187
1l. Ways to monitor/control caloric intake	109	15	141	31	0.220
1m. Benefits to fasting/intermittent fasting	107	17	136	36	0.148
1n. Impacts of organic vs. regular food	102	22	154	18	0.102
1o. Ways to control portion size	111	13	150	22	0.672
1p. Food labels to guide dietary decisions	119	5	162	10	0.674
**2. Vitamins, Minerals, and Supplements**					
2a. Glucosamine	63	57	67	96	0.075
2b. Vitamin D	56	64	87	76	0.320
2c. Omega 3 Fatty Acids	59	61	89	74	0.433
2d. Calcium	71	49	102	61	0.647
2e. Chondroitin	81	39	100	63	0.347
2f. Amino Acids	81	39	122	41	0.221
2g. Dietary Fiber	91	29	126	37	0.884
**3. Foods and Nutrients that may Reduce Inflammation**					
3a. Spices and Herbs	38	85	59	108	0.506
3b. Fruits and Vegetables	53	70	66	101	0.624
3c. Nuts	78	45	97	70	0.426
3d. Olive Oil	82	41	112	55	1.000
3e. Green Tea	95	28	105	62	**0.013**
3f. Fish Oil	86	37	132	35	0.101
3g. Plant-Based Alternatives for Meat	83	40	135	32	**0.014**
**4. Diets for Weight Loss**					
4a. Mediterranean Diet	51	69	82	84	0.301
4b. Low Carbohydrate Diet	74	46	76	90	**0.011**
4c. Fasting and Intermittent Fasting	69	51	98	68	0.890
4d. High Protein Diet	75	45	101	65	0.872
4e. DASH Diet	91	29	114	52	0.233
4f. Ketogenic Diet	98	22	110	56	**0.006**
4g. Vegetarian or Vegan Diets	83	37	143	23	**0.001**

Notes: LBMI denotes the lower Body Mass Index group (BMI < 30 kg/m^2^); HBMI denotes the higher Body Mass Index group (BMI > 30 kg/m^2^). Bold *p*-values denote statistical significance.

**Table 4:** Most commonly reported participant responses to the open-ended questions for each topic domain. Data are presented as *n* (%), and % indicates percentage of number of responses for the individual column for each question (i.e., % of entire cohort responses, % of group with higher BMI responses, and % of group with lower BMI responses). All responses can be found in Supplementary Materials (Table S1).

Survey Topic Domain and Response	Entire Cohort (*n* = 296)	BMI: <30 kg/m^2^; *n* = 124	BMI: ≥30 kg/m^2^; *n* = 172
**1. Weight Management and Healthy Lifestyle Topics**	**10 responses**	**4 responses**	**6 responses**
Reduce sugar cravings	1 (10%)	1 (25%)	-
Foods with natural anti-inflammatory properties	1 (10%)	1 (25%)	-
Alcohol and OA	1 (10%)	1 (25%)	-
Easy meals and snacks for increased energy	1 (10%)	1 (25%)	-
Treatment for eating disorders	3 (30%)	-	3 (50%)
Easy-to-prepare meals	1 (10%)	-	1 (17%)
Losing weight while managing multiple health issues	1 (10%)	-	1 (17%)
Medications that cause weight gain	1 (10%)	-	1 (17%)
**2. Vitamins, Minerals, and Supplements**	**20 responses**	**11 responses**	**9 responses**
Collagen	2 (10%)	2 (18%)	-
THC	2 (10%)	2 (18%)	-
CBD	2 (10%)	2 (18%)	-
Hyaluronic Acid	2 (10%)	2 (18%)	-
B Vitamins	3 (15%)	-	3 (25%)
OTC Pain Relief	2 (10%)	-	2 (17%)
**3. Foods and Nutrients that Reduce Inflammation**	**5 responses**	**2 responses**	**3 responses**
Chocolate	1 (20%)	1 (50%)	-
Alternate foods if you have intestinal issues	1 (20%)	1 (50%)	-
Butter substitutes	1 (20%)	-	1 (33%)
Shellfish	1 (20%)	-	1 (33%)
Tart Cherry	1 (20%)	-	1 (33%)
**4. Diets for Weight Loss**	**10 responses**	**3 responses**	**7 responses**
MIND	1 (10%)	1 (33%)	-
Weight Watchers	1 (10%)	1 (33%)	-
Reduce sugar	1 (10%)	1 (33%)	-
Diets to improve health condition	3 (33%)	-	3 (43%)
Balanced eating plan	3 (33%)	-	3 (43%)
Southern recipes	1 (10%)	-	1 (14%)
**5. Other Nutrition Topics Related to Joint Health You Would like to Learn About?**	**35 responses**	**10 responses**	**25 responses**
Foods to manage inflammation and pain	3 (9%)	3 (30%)	-
Diets for joint health in those who deal with other chronic health conditions (IBS, GERD, etc.)	3 (9%)	3 (30%)	-
Foods to prevent joint deterioration/ improve joint health	3 (9%)	1 (10%)	2 (8%)
Various foods on joint health (e.g., Asian diet, bone broth, white flour, Coke, Diet colas)	6 (18%)	-	6 (24%)
Foods to manage inflammation and pain	6 (18%)	-	6 (24%)
Foods that contribute to increased weight and pain	2 (6%)	-	2 (8%)
Nutritional choices can help for low density bones and prevention of fractures	2 (6%)	-	2 (8%)
**6. What other resources about joint health and nutrition you would you like?**	**21 responses**	**7 responses**	**14 responses**
Review articles—evidence-based information	7 (33%)	3 (43%)	4 (29%)
Appointments with other specialists/support group (rheumatologist, post gastric bypass specialist, eating disorder counseling, nutritionist, support groups)	6 (29%)	-	6 (43%)
**7. Summary of additional information noted but not related to nutrition.**	**25 responses**	**13 responses**	**12 responses**
Additional non-medical treatments (physical therapy, exercise, massage, etc.)	6 (24%)	6 (46%)	-
Relationship between OA and PCOS	2 (8%)	2 (15%)	-
Exercise and joint mobility (with joint pain)	2 (8%)	-	2 (15%)
Is losing weight fast to reduce stress on joints better than losing weight slowly and nutritiously?	2 (8%)	-	2 (15%)
**8. Irrelevant Responses**	**15 responses**	**6 responses**	**9 responses**
Responses that were given but deemed not relevant.	15 (100%)	6 (100%)	9 (100%)

## Data Availability

The raw data supporting the conclusions of this article will be made available by the authors on request due to restrictions within the approved IRB protocol.

## Supplementary Materials

The following supporting information can be downloaded at: https://www.mdpi.com/article/10.3390/nu17132056/s1, Figure S1: Bar graphs and unadjusted logistic regression p-value results for the proportion of those who reported interest for learning about each Strategy for Weight Management and a Healthy Lifestyle topic based on dichotomous BMI subgroup; Figure S2: Bar graphs and unadjusted logistic regression *p*-value results for the proportion of those who reported interest for learning about each General Information on Vitamins, Minerals, and Supplements topic based on dichotomous BMI subgroup; Figure S3: Forest plot summary of the unadjusted logistic regression models with continuous BMI as the explanatory variable for determining the odds of reporting interest in learning about General Information on Vitamins, Minerals, and Supplements topics of interest; Figure S4: Bar graphs and unadjusted logistic regression p-value results for the proportion of those who reported interest for learning about each Foods and Nutrients that may Reduce Inflammation topic based on dichotomous BMI subgroup; Figure S5: Bar graphs and unadjusted logistic regression p-value results for the proportion of those who reported interest for learning about each Diets for Weight Loss topic based on dichotomous BMI subgroup; Table S1: Participant Responses to the Open-Ended Questions for Each Topic Domain.

## Abbreviations

The following abbreviations are used in this manuscript:

OA	Osteoarthritis
BMI	Body Mass Index
HBMI	Higher Body Mass Index (dichotomized group)
LBMI	Lower Body Mass Index (dichotomized group)
OAAA	Osteoarthritis Action Alliance
OR	Odds Ratio
CBD	Cannabidiol
THC	Tetrahydrocannabinol
