# Supplementary material for: BMI-Specific Nutritional Education Priorities for Weight Management in Osteoarthritis
Source: Nutrients. 2025 Jun 20;17(13):2056. doi: 10.3390/nu17132056 (PMC12250860; doi:10.3390/nu17132056)
Supplement: Supplementary file 1 [file nutrients-17-02056-s001.zip › nutrients-3685529-supplementary.pdf]

Supplemental Figure S1. Bar graphs and unadjusted logistic regression p-value results for the proportion of those who reported interest for learning about each Strategy for Weight Management and a Healthy Lifestyle topic based on dichotomous BMI subgroup (Lower BMI: <30 kg/m^2; Higher BMI: ≥30 kg/m^2). Bold text and p-values represent a statistically significant result from the unadjusted logistic regression models with dichotomous BMI as the explanatory variable.

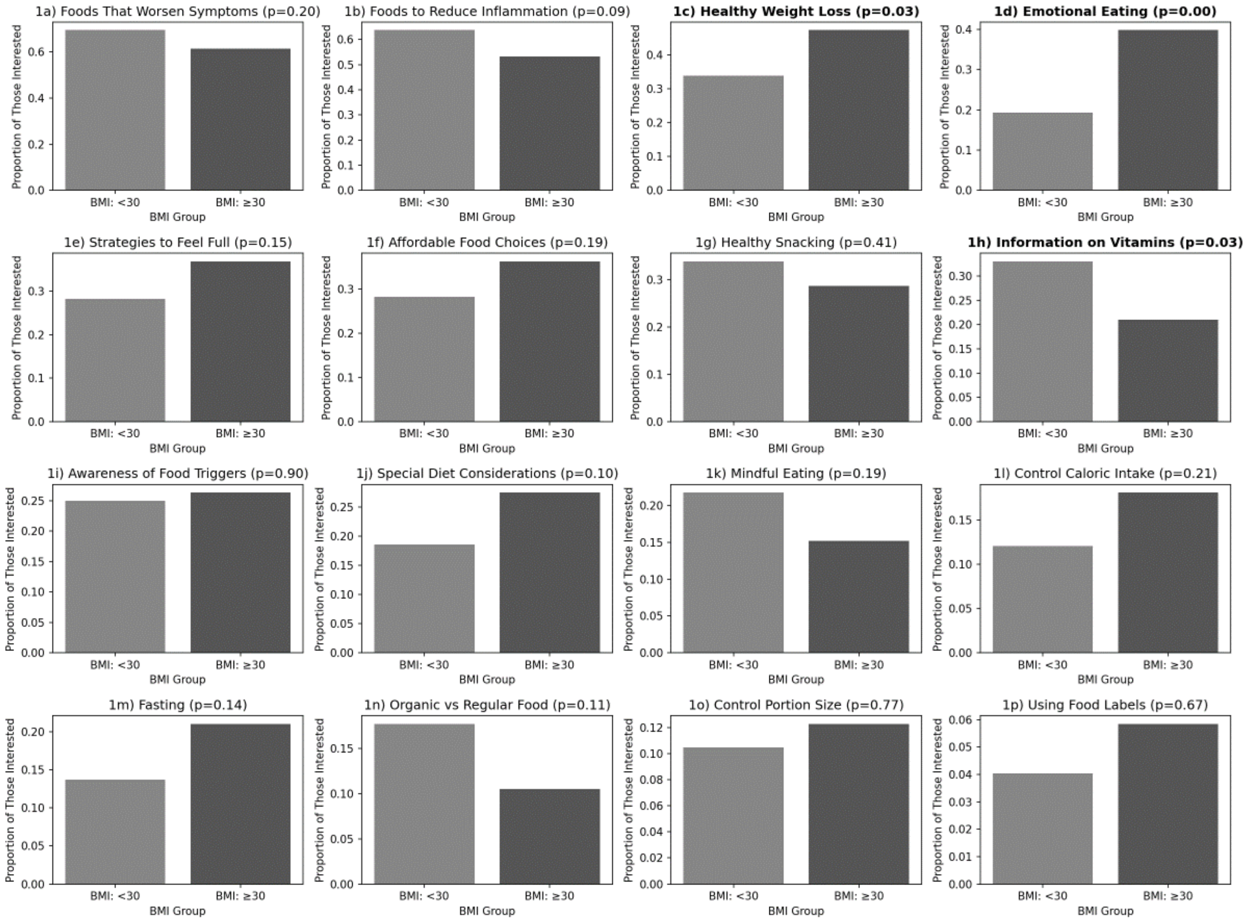

Supplemental Figure S2. Bar graphs and unadjusted logistic regression p-value results for the proportion of those who reported interest for learning about each General Information on Vitamins, Minerals, and Supplements topic based on dichotomous BMI subgroup (Lower BMI: <30 kg/m^2; Higher BMI: ≥30 kg/m^2). Bold text and p-values represent a statistically significant result from the unadjusted logistic regression models with dichotomous BMI as the explanatory variable.

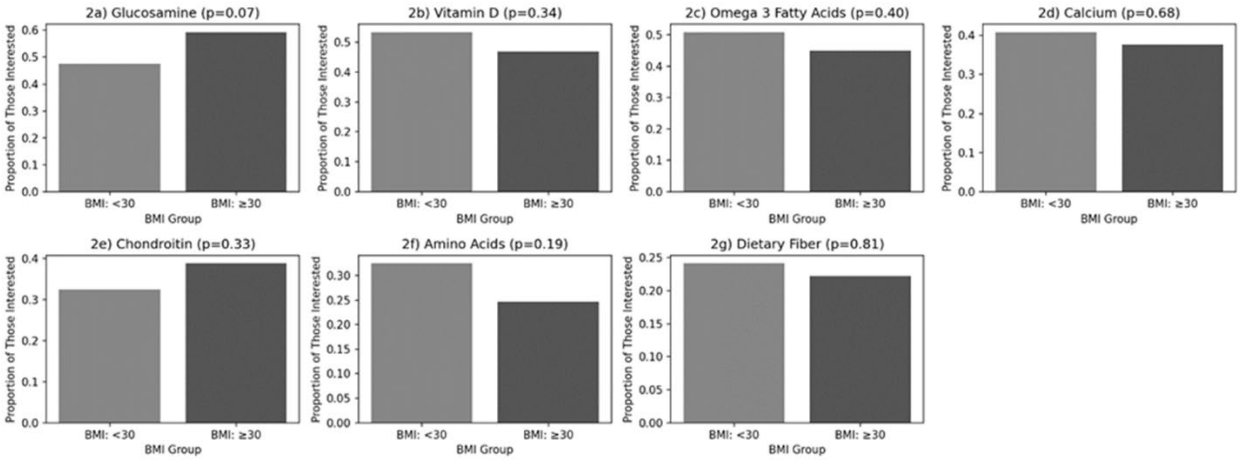

Supplemental Figure S3. Forest plot summary of the unadjusted logistic regression models with continuous BMI as the explanatory variable for determining the odds of reporting interest in learning about General Information on Vitamins, Minerals, and Supplements topics of interest. Results reported as odds ratio [95% confidence interval], and bold text indicates a statistically significant model (95% CI does not span 0).

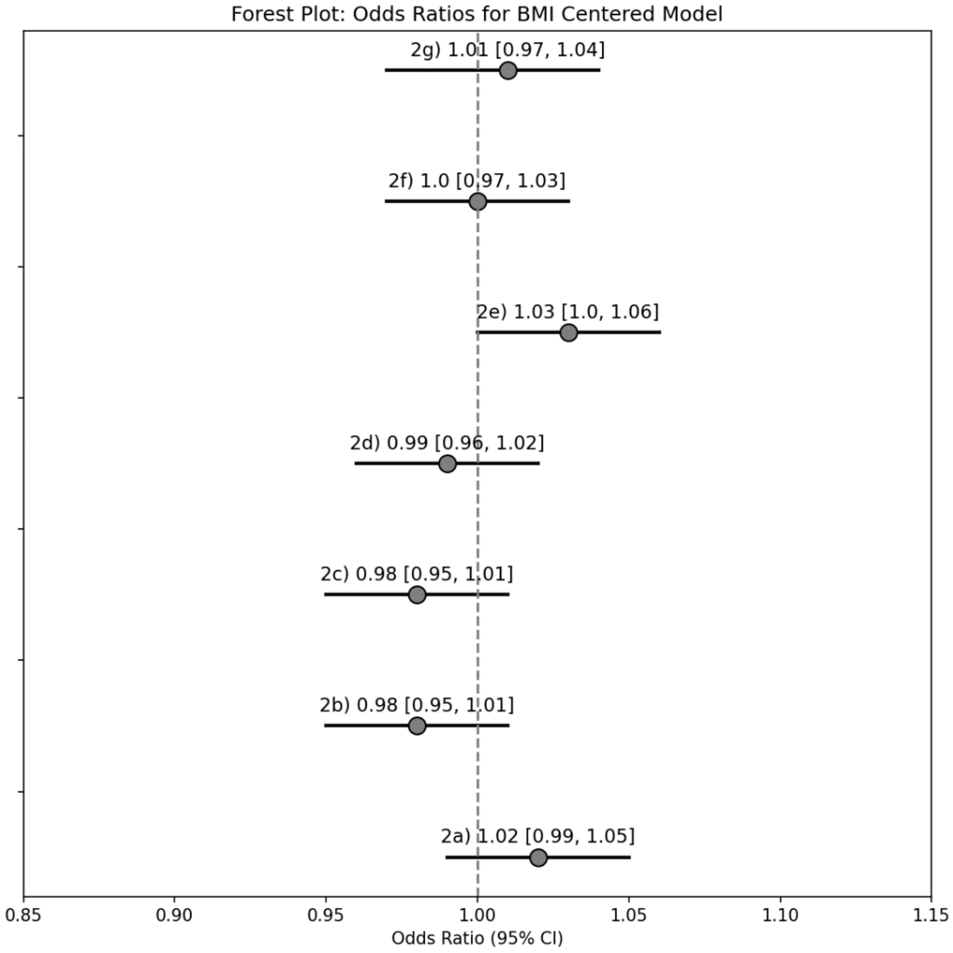

Supplemental Figure S4. Bar graphs and unadjusted logistic regression p-value results for the proportion of those who reported interest for learning about each Foods and Nutrients that may Reduce Inflammation topic based on dichotomous BMI subgroup (Lower BMI: <30 kg/m^2; Higher BMI: ≥30 kg/m^2). Bold text and p-values represent a statistically significant result from the unadjusted logistic regression models with dichotomous BMI as the explanatory variable.

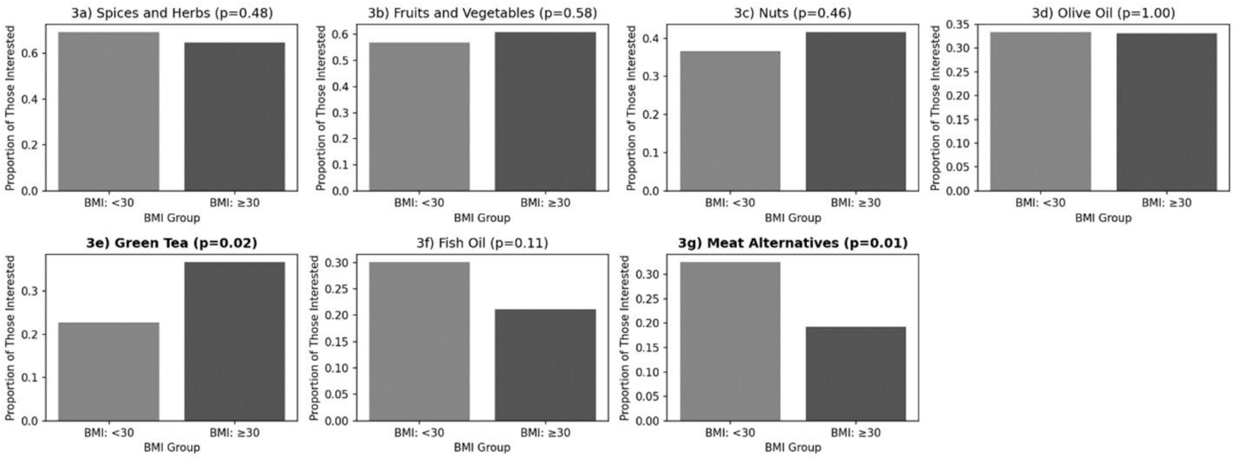

Supplemental Figure S5. Bar graphs and unadjusted logistic regression p-value results for the proportion of those who reported interest for learning about each Diets for Weight Loss topic based on dichotomous BMI subgroup (Lower BMI: <30 kg/m^2; Higher BMI: ≥30 kg/m^2). Bold text and p-values represent a statistically significant result from the unadjusted logistic regression models with dichotomous BMI as the explanatory variable.

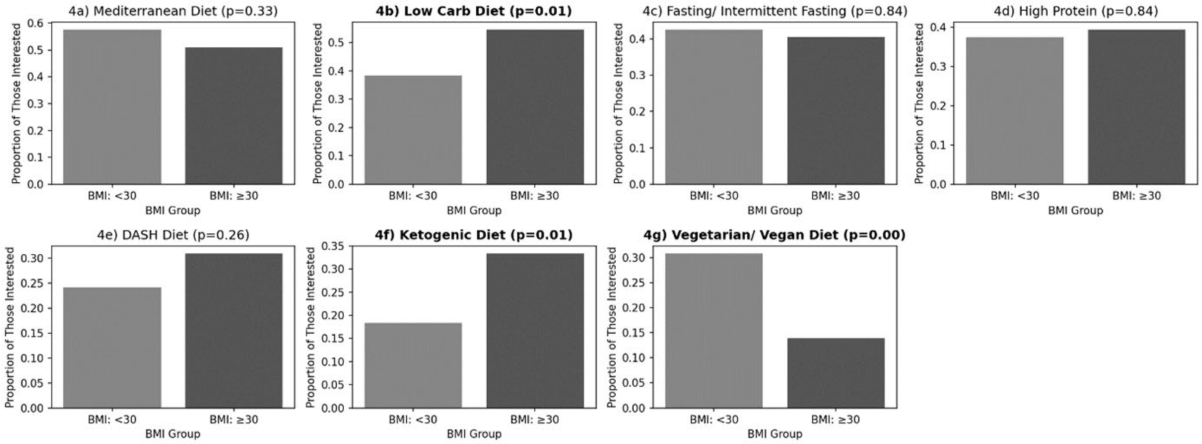

Supplemental Table S1. Participant Responses to the Open-Ended Questions for Each Topic Domain. Data are presented as n (%), and % indicates percentage of number of responses for the individual column for each question (i.e., % of entire cohort responses, % of group with higher BMI responses, and % of group with lower BMI responses).

Survey Topic Domain and Response	Entire Cohort (N=296)	BMI: <30 kg/m^2; n=124	BMI: ≥30 kg/m^2; n=172
1. Weight Management and Healthy Lifestyle Topics	10 responses	4 responses	6 responses
Reduce sugar cravings	1 (10%)	1 (25%)	-
Foods with natural anti-inflammatory properties	1 (10%)	1 (25%)	-
Alcohol and OA	1 (10%)	1 (25%)	-
Easy meals and snacks for increased energy	1 (10%)	1 (25%)	-
Treatment for eating disorders	3 (30%)	-	3 (50%)
Easy-to-prepare meals	1 (10%)	-	1 (17%)
Losing weight while managing multiple health issues	1 (10%)	-	1 (17%)
Medications that cause weight gain	1 (10%)	-	1 (17%)
2. Vitamins, Minerals, and Supplements	20 responses	11 responses	9 responses
Collagen	2 (10%)	2 (18%)	-
THC	2 (10%)	2 (18%)	-
CBD	2 (10%)	2 (18%)	-
CO-Q (antioxidant)	1 (5%)	1 (9%)	-
Hyaluronic Acid	2 (10%)	2 (18%)	-
Kratom	1 (5%)	1 (9%)	-
MSM	1 (5%)	1 (9%)	-
B Vitamins	3 (15%)	-	3 (25%)
OTC Pain Relief	2 (10%)	-	2 (17%)
Turmeric	1 (5%)	-	1 (8%)
Zinc	1 (5%)	-	1 (8%)
Calcium	1 (5%)	-	1 (8%)
Multivitamin	1 (5%)	-	1 (8%)

3. Foods and Nutrients that Reduce Inflammation	5 responses	2 responses	3 responses
Chocolate	1 (20%)	1 (50%)	-
Alternate foods if you have intestinal issues	1 (20%)	1 (50%)	-
Butter substitutes	1 (20%)	-	1 (33%)
Shellfish	1 (20%)	-	1 (33%)
Tart Cherry	1 (20%)	-	1 (33%)
4. Diets for Weight Loss	**10 responses**	**3 responses**	**7 responses**
MIND	1 (10%)	1 (33%)	-
Weight Watchers	1 (10%)	1 (33%)	-
Reduce sugar	1 (10%)	1 (33%)	-
Diets to improve health condition	3 (33%)	-	3 (43%)
Balanced eating plan	3 (33%)	-	3 (43%)
Southern recipes	1 (10%)	-	1 (14%)
5. Other Nutrition Topics Related to Joint Health You Would like to Learn About?	**35 responses**	**10 responses**	**25 responses**
Foods to manage inflammation and pain	3 (9%)	3 (30%)	-
Diets for joint health in those who deal with other chronic health conditions (IBS, GERD, etc.)	3 (9%)	3 (30%)	-
Foods to prevent joint deterioration/ improve joint health	3 (9%)	1 (10%)	2 (8%)
How food additives can affect arthritis.	1 (3%)	1 (10%)	-
Reducing red meat to manage joint pain	1 (3%)	1 (10%)	-
Various foods on joint health (e.g., Asian diet, bone broth, white flour, Coke, Diet colas)	6 (18%)	-	6 (24%)
Foods to manage inflammation and pain	6 (18%)	-	6 (24%)
Foods that contribute to increased weight and pain	2 (6%)	-	2 (8%)
Iron sources for vegans	1 (3%)	1 (10%)	-
Nutritional choices can help for low density bones and prevention of fractures	2 (6%)	-	2 (8%)
Diet fads to avoid	1 (3%)	-	1 (4%)

Nutrition for stage 4 kidney disease and joint health combined	1 (3%)	-	1 (4%)
Grocery shopping while on SNAP	1 (3%)	-	1 (4%)
Eating Disorder identification and treatment	1 (3%)	-	1 (4%)
How food additives can affect arthritis.	1 (3%)	-	1 (4%)
Healthy sweets	1 (3%)	-	1 (4%)
Reduce sugar cravings.	1 (3%)	-	1 (4%)
6. What other resources about joint health and nutrition you would you like?	21 responses	7 responses	14 responses
Audiobooks	1 (5%)	1 (14%)	-
Podcasts	1 (5%)	1 (14%)	-
Review articles – evidence-based information	7 (33%)	3 (43%)	4 (29%)
Combinations of diets for OA	1 (5%)	1 (14%)	-
Food allergies and diet related to weight loss.	1 (5%)	1 (14%)	-
Appointments with other specialists/support group (rheumatologist, post gastric bypass specialist, eating disorder counseling, nutritionist, support groups)	6 (29%)	-	6 (43%)
Participate in a research study	1 (5%)	-	1 (7%)
Texting	1 (5%)	-	1 (7%)
Information about the gut-mind connection	1 (5%)	-	1 (7%)
Getting resources on a limited income	1 (5%)	-	1 (7%)
7. Summary of additional information noted but not related to nutrition.	25 responses	13 responses	12 responses
Additional non-medical treatments (physical therapy, exercise, massage, etc.)	6 (24%)	6 (46%)	-
Relationship between OA and PCOS	2 (8%)	2 (15%)	-
How to increase ATP to help mitochondria with joint repair in cells where cytokines are causing healing not to occur in arthritic areas	1 (4%)	1 (8%)	-

Joint pain with fibromyalgia; complementary medicine	1 (4%)	1 (8%)	-
What causes the most problems to people with OA	1 (4%)	1 (8%)	-
Allergies as possible triggers	1 (4%)	1 (8%)	-
Gout	1 (4%)	1 (8%)	-
What supplements are ok to take with my other medications	1 (4%)	-	1 (8%)
Exercise and joint mobility (with joint pain)	2 (8%)	-	2 (15%)
Cartilage replacement	1 (4%)	-	1 (8%)
Cooking for one	1 (4%)	-	1 (8%)
The roles of metabolism and hormones on weight and joint health	1 (4%)	-	1 (8%)
Thyroid problem, Cystic obesity, Parkinson's, Fibromyalgia, and RA	1 (4%)	-	1 (8%)
Walking exercises that are easy on the joints	1 (4%)	-	1 (8%)
Is losing weight fast to reduce stress on joints better than losing weight slowly and nutritiously?	2 (8%)	-	2 (15%)
What supplements are ok to take with my other medications?	1 (4%)	-	1 (8%)
Exercising with joint pain in knee and lower back.	1 (4%)	-	1 (8%)
8. Irrelevant Responses	15 responses	6 responses	9 responses
Responses that were given but deemed not relevant.	15 (100%)	6 (100%)	9 (100%)
